# The utilization of the Vezzoni modified Badertscher distension device in breeding programs: Heritability estimates and effect on the hip dysplasia prevalence

**DOI:** 10.1371/journal.pone.0308984

**Published:** 2024-08-20

**Authors:** Birgit Deboutte, Louis Vandekerckhove, Emmelie Stock, Kaatje Kromhout, Anthony Morin, Jimmy H. Saunders, Luc Peelman, Bart J. G. Broeckx

**Affiliations:** 1 Department of Veterinary and Biosciences, Faculty of Veterinary Medicine, Ghent University, Merelbeke, Belgium; 2 Department of Veterinary Morphology, Imaging, Orthopedics, Rehabilitation and Nutrition, Faculty of Veterinary Medicine, Ghent University, Merelbeke, Belgium; 3 Breeding and Canine Development, CESECAH, Clermont-Ferrand, France; Human Genetics and Genome Research Institute, National Research Centre, EGYPT

## Abstract

Canine hip dysplasia (CHD) is a common orthopedic condition, influenced by both genetic and environmental factors. While current breeding programs often rely on ventrodorsal hip-extended (VDHE) radiographs, it is known they fail to accurately assess hip joint laxity. Therefore additional laxity-oriented diagnostic techniques have been developed. This study aims to evaluate the effectiveness of the Vezzoni modified Bädertscher distension device (VMBDD) technique, which quantifies hip joint laxity with the laxity index (LI), as a screening tool in two breeding programs. Data from a Belgian population of assistance dogs (population A) and a French population of guide dogs (population B) were analyzed. The heritability estimates of the LI, estimated using Bayesian statistical methods, were high in both populations (0.83 in population A and 0.82 in population B). Improved screening of parents by combining the VMBDD technique with the VDHE, significantly decreased LI and the prevalence of CHD in their offspring. In population A, when two parents were screened with the VMBDD compared to one, there was an average LI decrease of 0.03 (P<0.05). In population B, when one or both parents were screened with the VMBDD compared to none, the average LI decrease was 0.04 (P< 0.05) and 0.05 (P<0.01), respectively. In population A, screening both parents with the VMBDD in addition to the VDHE, resulted in 82.7% (P< 0.05) lower odds of CHD compared to screening only one parent. In population B, screening one parent led to 72.3% (P<0.05) lower odds of CHD compared to none of the parents being screened. In population B, when both parents were screened with the VMBDD, not a single case of CHD was observed in the puppies. In conclusion, based on these results, the VMBDD technique has the potential to drastically reduce CHD prevalence and is as such an excellent tool for breeding programs.

## Introduction

Canine hip dysplasia (CHD) is a common, debilitating condition in many dog breeds. As many orthopedic disorders, it is a multifactorial disease, implying that the hip joint phenotype is the resultant of the combined effect of genetic and environmental contributors [1–4]. While various efforts have been undertaken to reduce its prevalence by selective breeding, success has been limited, with the exception of a few breeding programs [5]. Nevertheless, even in the more successful programs, a redirection from the classical grading schemes that use the standard ventrodorsal hip-extended (VDHE) radiographs, to a more laxity-oriented view, based on specific techniques capable to quantify hip joint laxity, has been proposed [5–11].

From a pathogenesis point of view, laxity is key in the development of CHD. In more detail, already decades ago, it has been hypothesized and confirmed later on that hip joint laxity, defined as “the amount of movement of the femoral head in the acetabulum”, influences the probability to develop osteoarthritis (OA) later on in life [1]. Unfortunately, due to the positioning of the dog, the traditional VDHE radiographs fail to sufficiently visualize hip joint laxity [1, 2, 12]. This is the reason why various laxity-oriented diagnostic techniques have been developed. While their superiority for diagnostics and screening purposes has been demonstrated in several studies, it is ultimately their added value in breeding programs that will make it possible to work towards a final solution, i.e. the elimination of CHD from dog populations [5, 8, 11, 13]. Unfortunately, data for that purpose, is scarce and, to the authors’ knowledge, even non-existent for the technique studied here.

In more detail, recently, the technical aspects, the laxity measurements and the bio-mechanical properties of the coxofemoral joints with regards to one specific laxity-oriented screening technique, the Vezzoni modified Bädertscher distension device (VMBDD) technique, which expresses laxity as the “laxity index” (LI), have been further studied [6–11]. The main goal of the current study is to evaluate the effectiveness of the VMBDD technique as a screening tool in breeding programs. This was done in two steps.

Before implementing a certain trait (phenotype) in a breeding program, it is important to determine how well it will respond to selection. An indication of what to expect, can be based on the (narrow sense) heritability h^2^, which is defined as the proportion of the total variability of a trait attributable to the additive genetic variance. There will be little resemblance between relatives and little response to selection when a trait has a low h^2^ compared to a trait with high h^2^ (if the intensity of selection and the variability of the phenotype is the same)[14]. So ideally, we want the trait under selection to have a high h^2^. Narrow sense heritability h^2^ estimates are estimates linked to a specific phenotype but also to a specific population as it depends e.g. on the variability of the phenotype and also on the environment in that population [14]. Because of these characteristics, the first aim was to estimate h^2^ and this was done in two different populations, to have a rough idea on the stability of the estimate.

The two populations in question are both breeding programs where the breeding stock is selected based primarily on their own phenotypic values. This is called ‘individual selection’ or ‘mass selection’, where the individuals with the most desirable phenotypes are selected. This is the simplest method of selection and is also used frequently [14]. As such, the second goal was to evaluate the influence of including the VMBDD technique and LI as a screening technique in these breeding programs.

## Materials and methods

### Data collection

Data from two breeding programs was retrospectively collected. One breeding program (population A) was a Belgian breeding program for assistance dogs. The second breeding program (population B) was located in France and specifically focuses on guide dogs. In both programs, the hips of the dogs were screened with VDHE radiographs and for a subset of the dogs, the VMBDD technique was also used to quantify laxity in the left and the right hip joints. In population A, the LI was measured around the age of 1 year for all dogs born in this breeding program. In population B, the LI was also measured around the age of 1 year, but only for the dogs selected at 8 weeks as potential breeders. Both breeding programs use the Fédération Cynologique Internationale (FCI) grading system to evaluate the VDHE radiograph. All radiographical screening and evaluations were executed at the veterinary practices that routinely perform these procedures for the respective organizations.

### h^2^ estimates

The narrow sense heritability h^2^ was estimated using Bayesian statistical methods, as described by Gianola and Sorensen [15]. A likelihood ratio test was used to evaluate if there was a significant difference in LI between sex or between left and right hip. LI was then modeled as a function of sex (after it was established that there was a significant difference in LI between sexes) and the additive genetic contribution, in the form of the following linear mixed model: *LI*_*i*_ = *β*_0_+*β*_*sex*_*sex*_*i*_+*u*_*a*_*a*_*i*_+*e*_*i*_, where *β*_0_ is an unknown constant common to all dogs, *sex*_*i*_ indicates the *ith* dog’s sex (male/female) as a fixed predictor with *β*_*sex*_ the coefficient for sex, *a*_*i*_ indicates the additive genetic contribution for the *ith* dog as a random predictor, with *u*_*a*_ as a coefficient and *e*_*i*_ is the unknown residual for the *ith* dog. The analysis was performed in R with the “MCMCglmm”-package [16]. This package uses a Markov Chain Monte Carlo (MCMC) Gibbs sampling algorithm to fit the models. For the fixed effects the default normal distribution prior is used with mean zero and large variance. The package has a built-in inverse-Wishart prior for the variance structures of the random effects (*V*_*a*_) and the residuals (*V*_*e*_). The initial sampling value for both *V*_*e*_ and *V*_*a*_ was set to half the phenotypical variance and the degrees of freedom parameter was set at 0.002, which resulted in flat priors. This translates to a prior that specifies that phenotypical variance is equally influenced by genetic and residual control, but as it is a non-informative prior, this also implies that this initial specification basically has close to no effect as soon as the data says otherwise. To account for the relationships between relatives, the package relies on the inverse of the numerator relationship matrix A^-1^, as described by Henderson [17]. A^-1^ is calculated based on a separately provided pedigree of the animals included in the analysis. Convergence of the posterior densities was evaluated through trace plots and the degree of autocorrelation among the samples. The h^2^ was then estimated as mode of the posterior distribution of *h*^2^ = *V*_*a*_/(*V*_*a*_+*V*_*e*_) [14].

### The effect of screening parents with a laxity-based technique on the LI and CHD prevalence of the offspring

To evaluate the effect of the number of parents (zero, one or two parents screened with the VMBDD) on the LI of offspring, a linear regression model was constructed with LI of the offspring as the response variable and the sex and the number of parents that had known LI as the predictor variables. The same was done for CHD diagnosis, but a logistic regression model was used with CHD diagnosis as a binary response variable (healthy/dysplastic). Dogs with an FCI grading of ‘A’ or ‘B’ were considered healthy, dogs with an FCI grading of ‘C’, ‘D’ or ‘E’ were diagnosed with CHD. In all regression models, significance was evaluated using the likelihood ratio test.

## Results

### Descriptive statistics

Two datasets of dogs were available. All dogs were Labrador retrievers, with laxity quantified with the LI, obtained with the VMBDD technique. The sample from population A contained 168 unique dogs and was provided by a Belgian breeding program for assistance dogs. In this group, the mean LI was 0.33 and LI ranged between 0.03 and 0.73 and had a standard deviation of 0.11. The second dataset (from population B) was derived from a French guide dog breeding program and contained 117 dogs. For this sample, the mean LI was 0.24 and ranged between 0.03 and 0.58 and had a standard deviation of 0.11.

In population A, 56 dogs had only one parent with a known LI and for 87, the LI of both parents was available. For 25 dogs, the screening method of the parents was unknown, these were excluded from the regression analysis. In population B, the LI of the parents was unknown for 47 dogs, was available for one parent for 48 dogs, and in both parents for 22 dogs. Population A had 5 dogs diagnosed with CHD, the other 163 dogs had healthy hips. Eight dogs from population B were diagnosed with CHD, 109 were healthy. The dogs in population A were born between 2018 and 2023, the dogs in population B were born between 2014 and 2021. Of the dogs in population A, 73 were female and 95 were male, in population B 89 were female and 28 were male.

### h^2^ estimates

In both samples, a significantly higher LI was seen in female compared to male dogs (population A: mean difference = 0.05 (P < 0.001), population B: mean difference = 0.07 (P < 0.001), respectively). There was no significant difference in LI between the right and left hip joint in neither of the samples (population A: mean difference = 0.01 (P = 0.44), population B: mean difference = 0.01 (P = 0.39), respectively). Sex was included as a fixed effect in the linear mixed model but not the side of the hip joint. In the estimation of the h^2^, the posterior distributions showed convergence and a low degree of autocorrelation in both samples. For population A, the estimated h^2^ was 0.83 (95% high density interval (95HDI): [0.78; 0.89]), in population B, the h^2^ was estimated to be 0.82 (95HDI: [0.74;0.88]), respectively. An overview of the estimates is provided in Table 1.

**Table 1:** A summary of the results for each of the populations: difference in laxity index (LI) between sex, difference in LI between hip side, heritability (h^2^) of LI and odds ratio of CHD in offspring when 0/1/2 parents are screened for LI.

	OLS/Bayes Estimate*	95% CI/ 95%HDI*	p-value
**Population A** **			
LI female compared to male	0.05	[0.04;0.07]	< 0.001
LI left compared to right	0.01	[-0.01; 0.03]	0.43
h^2^ of LI	0.83	[0.78;0.89]	-
Difference in LI in offspring when: 1 parent screened with VMBDD and			
2 parents screened	-0.03	[-0.05;0]	< 0.05
Odds ratio of having CHD in offspring when 2/1 parents screened with VMBDD:			
2 parents / 1 parents	0.17	[0.03;0.71]	< 0.05
**Population B** ***			
LI in female compared to male	0.07	[0.04; 0.10]	< 0.001
LI left compared to right	0.01	[0; 0.03]	0.389
h^2^ of LI	0.83	[0.75;0.88]	-
Difference in LI in offspring when: 0 parents screened with VMBDD and			
1 parent screened	-0.04	[-0.05;-0.02]	< 0.05
2 parents screened	-0.05	[-0.07;-0.03]	< 0.01
Odds ratio of CHD in offspring when 1/0 parents screened with VMBDD:			
1 parents / 0 parents	0.28	[0.08, 0.84]	< 0.05

*Estimates are obtained through OLS with a corresponding 95% confidence interval, except for the heritability estimates, which are the mode of the posterior distribution of *h*^2^ = *V*_*a*_/(*V*_*a*_+*V*_*e*_), with an accompanying 95%-high density interval.

** In population A, none of the dogs had 0 parents screened for LI, so no comparison was possible.

*** In population B, none of the offspring from two parents screened with the LI had CHD. OLS = ordinary least squares, HDI = high density interval, CHD = canine hip dysplasia, CI = confidence interval

### The effect of screening parents with a laxity-based technique on the LI and CHD prevalence of the offspring

**Fig 1:**
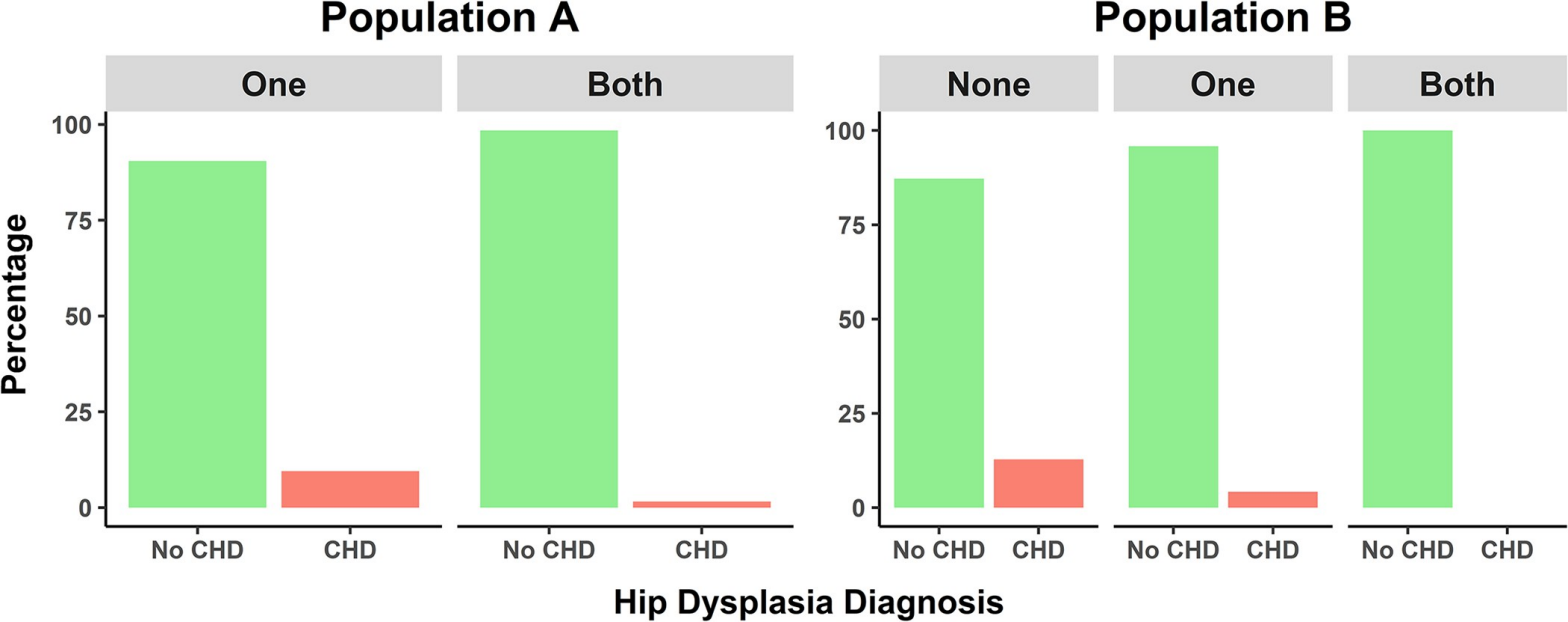
Difference in number of dogs diagnosed with CHD depending on the number of parents that were screened for hip laxity. There was a significant difference in number of puppies diagnosed with canine hip dysplasia (CHD) when the laxity index was known in none, one or both of their parents. In population A, the odds of being diagnosed with CHD were 83% lower for dogs where LI was known for both parents compared to when it was measured in only one parent. In population B, the odds of being diagnosed with CHD were 73% lower when LI was known for one parents compared to when it was known in none of the parents. None of the dogs who had two parents with a measured LI were diagnosed with CHD in this population.

The number of parents that were screened for hip laxity had a significant effect on the LI of the offspring (population A: P < 0.05, population B: P < 0.01), with an average decrease in the LI of 0.03 when two parents were screened compared to only one in population A and an average decrease in the LI of 0.04 and 0.05 when one and both parents, respectively, were compared to none of the parents screened in population B. There was a significant difference in number of dogs diagnosed with CHD depending on the number of parents that were screened for hip laxity in both populations (population A: P < 0.05, population B: P < 0.01) (Fig 1). In population A, it was estimated that when both parents are screened, the odds of CHD in their offspring are 82.7% lower than when only one parent is screened for laxity. In population A, there was no offspring where none of the parents had their LI measured.

Similarly, in population B, the estimated odds for CHD are 72.3% lower when one parent was screened for laxity compared to when none of the parents are screened. In this population, no CHD was diagnosed in the offspring from dogs where both parents had known LI. An overview of the data is presented in Table 1. Based on the observed LI difference between sexes, the probability to develop CHD was also compared between sexes: a significant difference was found in population A, where the odds of having CHD in females was 4.5 times the odds of having CHD in males (P < 0.05), but not in population B (odds ratio = 3.02, p = 0.11).

### Discussion and conclusion

CHD continues to be a problem in many dog populations, despite extensive research dedicated to improving screening methods and managing this disease in dog populations [2, 3, 5, 11, 13, 18–22]. Despite the development of additional screening methods, the VDHE radiograph still remains the primary screening technique, even though this method has several limitations. One such limitation is that the VDHE radiographic scoring techniques used in CHD assessment, such as those used by the FCI, the Orthopedic Foundation for Animals (OFA), or The British Veterinary Association and The Kennel Club (BVA/KC) are considerably influenced by the evaluator and expertise of the scoring veterinarian, raising concerns about its objectivity [18]. Simultaneously, a low h^2^ has been reported for these techniques [4, 19, 23]. Furthermore, they do not correctly quantify hip joint laxity and are sensitive to which sedation/anesthesia protocol is used [1, 2, 12].

Thus, it is important to base selection on phenotypes that are objective (defined as “not influenced by the opinion or expertise of an evaluator”), can be measured with high accuracy (which is defined as “a low systematic error” or a high “trueness” and a low random error or high “precision”, in ISO standard ISO5725-1) and have a high h^2^ [5, 14]. Laxity-based techniques have the advantage that they quantify the primary starting point of CHD, i.e. hip joint laxity, objectively [6, 8] and accurately [11]. Here, we wanted to evaluate whether this measurement fulfills the final requirement: a high h^2^.

Narrow sense heritability estimates have been reported for the PennHIP “Distraction Index” (DI) [5, 24], but no such study has been done for the LI. The h^2^ of a certain phenotype allows estimating to which extent that phenotype will respond to selection when selection pressure is applied. As h^2^ is based on the proportion of phenotypic variance (*V*_*p*_) that is due to additive genetic influence alone (additive genetic variance = *V*_*a*_) and these variances are population- and phenotype-specific, it makes sense that h^2^ estimates are as well. Genetic components are influenced by allelic frequencies in the population and the environmental variance may also differ between different populations. In this study, h^2^ was estimated in two different populations that consisted of Labrador retrievers. The estimates were nearly identical, which is of course positive compared to a situation where two entirely different estimates would have been found as it substantiates that the estimates are valid and not a one-off case. This does not imply however that these values are to be taken for granted. As explained earlier on, they are population- and characteristic-specific. In populations with e.g. a larger variability in LI, or a greater variability in environmental factors, h^2^ could be higher or lower. They do indicate however that hip joint laxity in the two populations studied here, is strongly genetically influenced. Leighton et al. already recommended selecting on hip joint laxity, quantified with the PennHIP-based DI, as the DI had a high h^2^ of 0.66 [5]. Likewise, our results demonstrate that the heritability of the LI, assessed with the VMBDD technique, is high, especially compared with the h^2^ estimates of the VDHE radiograph-based OFA grading system, which are around 0.2–0.3 [4]. Intuitively, choosing dogs based on LI would thus likely reduce the occurrence of CHD in the dog.

To further investigate this claim, LI was compared between offspring where both parents had a known LI, offspring where the LI was known for only one parent and where LI was known for no parents, respectively. The same was done for CHD diagnosis: is the number of parents with known LI associated with the number of offspring that had radiographical signs of CHD, as determined by the FCI grading system? This can be viewed as a very straightforward approach to demonstrate that prior selection based on LI was linked to a reduction in LI values and, concurrently, a decrease in CHD diagnoses in these populations. The results were clear: there was an obvious decrease in LI, and even more so in the odds to develop CHD. Practically, this also indicates that even screening one parent with the VMBDD can have a significant effect on reducing CHD prevalence. However, to obtain the best result, we recommend screening both parents.

Aside from the main results, the systematic occurrence of sex and side (left/right) differences were checked. A significant difference in laxity between female and male dogs was found. In both populations, female dogs showed a significantly higher LI than male dogs. This aligns with the observations made by Ginja et al. (2008) [24]. While limited information exists on this subject in dogs, studies conducted on humans have demonstrated that women tend to have greater hip joint laxity compared to men [25, 26]. This was an interesting observation, which also raises the question if female dogs are more prone to develop CHD than male dogs. Here, the results were population-dependent. While only significant in one population, the trend was however the same in both: female dogs had higher odds to develop CHD. In other studies, a sex predisposition is not consistently noticed [27, 28]. A similar analysis was done for the side of the hip joint, but no significant difference in laxity was found between the right and the left hip joint, which again aligned with the findings of Ginja et al. (2008) [24] with PennHIP. This was to be expected, as there is no basis to suspect a difference in development between the two hip joints.

One potential limitation of this study is that it was conducted in only one breed (although in two different populations). As already mentioned, inherent to the characteristics of the h^2^, h^2^ estimates cannot necessarily be extrapolated to other populations of Labradors or other breeds. Future studies on other breeds might as such be interesting.

In conclusion, we found high h^2^ estimates of the LI in the two populations examined in this study. This finding was substantiated by the observation that when the LI was used to select suitable breeders, a notable decrease was observed in both the LI of the offspring and the odds of them to develop CHD. Taking the high h^2^ and the demonstrated outcomes into account, combined with the other advantageous characteristics of the VMBDD, the inclusion of the VMBDD technique and the LI as a selection criterion to reduce the incidence of CHD in a population, can only be encouraged.

## Supporting information

S1 File Legend for supporting information S2–S5. (TXT)

S2 File Data from population A analyzed for this report. (CSV)

S3 File Data from population B analyzed for this report. (CSV)

S4 File Pedigree relationships among all dogs in population A. (CSV)

S5 File Pedigree relationships among all dogs in population B. (CSV)

S6 File R code for all data analysis performed in this article (R). (R)
